# Autophagy Inhibition Promotes 5-Fluorouraci-Induced Apoptosis by Stimulating ROS Formation in Human Non-Small Cell Lung Cancer A549 Cells

**DOI:** 10.1371/journal.pone.0056679

**Published:** 2013-02-18

**Authors:** Xiaohong Pan, Xiuli Zhang, Hongliu Sun, Jinjin Zhang, Miaomiao Yan, Huaibin Zhang

**Affiliations:** 1 Department of Pharmaceutical Sciences, Binzhou Medical University, Shandong Yantai, China; 2 Medical Research Center, Binzhou Medical University, Shandong Yantai, China; Osaka University Graduate School of Medicine, Japan

## Abstract

Chemotherapy is an important option for the treatment of various cancers including lung cancer. However, tumor resistance towards cytotoxic chemotherapy has become more common. It has been reported that autophagy is one of the processes contributing to this resistance. In the present study, we found that the anti-cancer drug 5-fluorouraci(5-FU) could induce autophagy in A549 cells. 5-FU treatment could lead to the conversion of LC3 I/II, the up-regulation of Beclin-1, the down-regulation of p62 and the formation of acidic vesicular organelles (AVOs) in A549 cells. Pre-treatment of cancer cells with 3-MA or siAtg7 could enhance 5-FU-induced apoptosis through the activation of caspases, and the caspase inhibitor z-VAD-fmk rescued the cell viability reduction. Furthermore, the inhibition of autophagy also stimulated ROS formation and scavenging of ROS by antioxidant NAC inhibited caspase-3 activity, prevented the release of cyt-c from mitochondria and eventually rescued cancer cells from 5-FU-mediated apoptosis. These results suggest that 5-FU-elicited autophagic response plays a protective role against cell apoptosis and the inhibition of autophagy could sensitize them to 5-FU-induced caspase-dependent apoptosis through the stimulation of ROS formation.

## Introduction

Lung cancer is one of the most common malignancies in the world and the leading cause of cancer-related death in many countries. Approximate 85% of lung cancer cases belong to non-small-cell lung cancer (NSCLC) [1], [2]. Chemotherapy is an important option in curing or controlling lung cancer. 5-fluorouracil (5-FU), which exerts its anticancer effects through the inhibition of thymidylate synthase and the incorporation of its active metabolites into RNA and DNA so as to influence the uracil metabolism and eventually lead to apoptosis in the cancer cell [3]. In the past decades, 5-FU-based combination therapies are standard treatments for many patients diagnosed with various malignant tumors, including NSCLC [4]–[6]. However, along with its usage, resistance to 5-FU has become common and has been recognised as a reason for many cancers therapy failure [7], [8]. Therefore, many attempts have been carried out in order to reduce the resistance and enhance its therapeutic effectiveness. Although many aggressive therapies, such as new drugs combined with 5-FU, have improving patients survival, the effect of these therapies remains far from satisfactory at present. It is consequently desirable to find more appropriate therapeutic opportunities for NSCLC. Herein, we report the induction of autophagy by 5-FU in human NSCLC A549 cells.

Over the past decades, apoptosis induction has been the major consideration in anti-cancer drug development. However, cancer cells trigger multiple pathways to escape from apoptosis [1]. Recently, autophagy has been widely studied in cancer therapy. In addition to its housekeeping role in removing misfolded or aggregated proteins, clearing damaged organelles and eliminating intracellular pathogens, autophagy has multiple physiological and pathophysiological functions in cancer therapy. Many studies have focused on the relationship between autophagy and tumour pathogenesis, development and treatment. However, autophagy seems to play a paradoxical role in cancer cell survival and death. In chemotherapy, when cells encounter some anti-cancer drugs, autophagy is induced to protect cancer cells against apoptosis for cell survival. Therefor autophagy is recognized as a cytoprotective process [7], [9]–[11]; Meanwhile, Recent studies have shown that the inhibition of autophagy induces lowered apoptotic level, therefore, autophagy participates in the upregulation of apoptosis [12], [13]; Furthermore, like apoptosis, autophagy is also an alternative route of programmed cell death, called type-II programmed cell death [14]–[16]. Presumably, the role of autophagy may depend on the type of tumor and stimuli, the stage of tumorigenesis and apoptotic status in tumor cells. Appropriate modification of autophagy, inhibition of cytoprotective autophagy to enhance the apoptosis of tumor cells in response to anti-cancer agents might improve the effects of chemotherapy [9]. Thus, in addition to apoptotic response, the study of autophagy is a prospective direction for the development of anti-cancer drugs.

Reactive oxygen species(ROS) play an important role in a variety of cellular programs during physiological as well as pathological conditions. When produced in moderate amounts, ROS act as signaling molecules in signal transduction pathways to regulate cell growth, differentiation, survival, inflammation and the immune response [17]. On the other hand, when excessively produced, they share the ability to inflict oxidative damage to vital biological molecules, like DNA, lipids and proteins, which alters their functionality and causes impairment of cellular integrity [18]. In the past years, mounting evidence indicates that ROS are implicated in autophagy induction in cancer therapy [19]–[21], suggesting that ROS play a crucial role in response to cancer therapeutics, deregulation of ROS formation is associated with cancer initiation, progression and drug resistance.

In this study, we investigated the mechanism underlying the anti-cancer effects of 5-FU in A549 lung carcinoma. We found that 5-FU induced autophagy in A549 cells and the inhibition of autophagy could lead to the enhancement of 5-FU-mediated apoptosis. Furthermore, we demonstrated the mechanism that autophagy inhibition sensitized cell to apoptotic cell death was by increasing the formation of ROS that eventually facilitated the release of cytochrome c from mitochondria, which subsequently enhanced caspase-dependent apoptosis.

## Materials and Methods

### Cell culture

The human NSCLC cells A549 were obtained from the Type Culture Collection of the Chinese Academy of Sciences(Shanghai, China). The cells were cultured with RPMI-1640 medium (Gibco, Carlsbad, CA, USA) supplemented with 10% foetal bovine serum and antibiotics (100 U/ml penicillin and 100 µg/ml streptomycin) at 37°C under a humidified atmosphere of 95% air and 5% CO_2_. Cells in the logarithmic phase of growth were used in this study.

### Chemical Reagents and antibodies

5-FU, 3-MA, 3-(4,5-dimethyl-2-thiazolyl)-2,5-diphnyl-2H-tetrazolium bromide (MTT), 5,5′,6,6′-tetrachloro-1,1′,3,3′-tetraethylbenzimidazolyl carbocyanine iodide (JC-1) and 5-(and 6)-carboxy-2′7′-dichlorodihydrofluorescein diacetate (DCFDA) were all purchased from Sigma-Aldrich (St. Louis, MO, USA). Anti-LC3, anti-Beclin1, anti-p62, anti-cytochrome c, anti-cleaved caspase9, anti-cleaved caspase8, anti-cleaved caspase3 and anti-cleaved PARP antibodies were purchased from Cell Signaling Technology (Danvers, MA, USA). Anti-actin antibody and the second antibodies were obtained from Santa Cruz Biotechnology(CA, USA).

### Cell viability assay

Cell viability was determined by MTT assay. Cells were seeded in 96-well flat bottom microtiter plates at a density of 1×10^4^ cells/ml with 100 µL per well, incubated for 24 h and then exposed to the indicated concentrations of 5-FU for the indicated times. After treatment, 20 µl MTT solution (5 mg/ml) was added to each well and incubated in a humidified 5% CO_2_ atmosphere at 37°C for 4 h. The crystals were then dissolved in 100 µl dimethyl sulfoxide/well. The absorbance of the solution was measured at 490 nm with a microtiter plate reader (Bio-Tek ELX800). Cell viability was calculated according to the following formula: Cell viability(%) = A490 (sample)/A490 (control)×100. At least three replicates were performed for each treatment.

### Immunofluorescence for LC3

The level of LC3 was examined using an immunofluorescence assay according to our previous report [22]. Briefly, A549 cells were seeded on glass coverslips. After treatment with 5-FU (10 µM) for 48 h in the presence or absence of 3-MA (5 mmol/L), cells were fixed with 4% paraformaldehyde for 15 min. After fixation, the cells were permeabilized with 0.5% Triton X-100 for 30 min and then blocked with 2% BSA for 1 h at room temperature. After blocking, cells were incubated with anti-LC3(1∶400 diluted in BSA buffer) antibody at 4°C overnight and then reacted with fluorescein isothiocyanate (FITC)-conjugated goat anti-rabbit IgG(1∶100 diluted in BSA buffer) for 1 h at 37°C. The nucleus were stained with 1 µmol/L DAPI (Sigma-Aldrich) for 5 min. Then LC3 puncta and stained nucleus were detected under a fluorescence microscope and merged(Olympus, Japan).

### Detection of acidic vesicular organelles (AVOs)

To quantify the development of AVOs, A549 cells were seeded in 24-well flat bottom microtiter plates at a density of 1×10^4^ cells/ml with 1 mL per well and incubated for 24 h. After treatment with 10 µM 5-FU for 48 h in the presence or absence of 3-MA (5 mmol/L), cells were stained with 1 µM acridine orange at 37°C in the dark for 15 min, then washed twice with PBS. Images of AO staining were visualized immediately using fluorescence microscope. To quantify the number of acidic vesicles in the treated cells, other cells were seeded into 6-well plates. After staining with AO in PBS at 37°C for 15 min, the cells were harvested, washed twice in PBS and resuspended in 200 µl PBS, then analyzed by flow cytometry assay. Green (500–550 nm, FL1 channel) and red (>650 nm, FL3 channel) fluorescence, which was illuminated with blue (488 nm) light excitation, was measured using flow cytometer with CellQuest analysis software (Becton Dickinson).

### Apoptosis detection by flow cytometry

The detection was performed by the AnnexinV-FITC Apoptosis Detection Kit(BD Biosciences, San Diego, USA). Breifly, Cells were seeded in 6-well plates and incubated for 24 h and then exposed to 10 µM 5-FU for 48 h in the presence or absence of 3-MA (5 mmol/L). After treatment, approximately 1×10^6^ cells were harvested, washed twice in PBS, and then stained with Annexin V-FITC and PI according to the manufacturer's instructions. The resulting fluorescence was detected by flow cytometry with CellQuest analysis software.

### Mitochondrial Membrane Potential (MMP) Analysis

The values of mitochondrial membrane potential were determined by flow cytometry using JC-1 staining according to the manufacturer's instructions. Briefly, the treated-cells were collected, washed with PBS, and incubated with 10 µM JC-1 for 30 min at 37°C in the dark. The positive cells were then detected by flow cytometer with CellQuest analysis software.

### Measurement of reactive oxygen species (ROS)

ROS levels were determined using the fluorescent marker 2′,7′-dichlorodihydrofluorescein diacetate (DCFH-DA). Briefly, the treated-cells were trypsinized, washed and incubated with 10 µM DCFH-DA for 30 min in the dark, and the intensity of fluorescence was followed by flow cytometer with CellQuest analysis software.

### Measurement of Cytochrome c Release

The mitochondria fraction and cytosol fraction was prepared by different centrifugation as Jie Li, MD described [3]. Briefly, the treated-cells were harvested and washed twice with PBS, then were resuspended in a sucrose buffer (250 mM sucrose, 1 mM EDTA, 50 mM Tris-HCl pH 7.5 supplemented with 1 mM PMSF, 1 µg/mL leupeptin, 1 µg/mL pepstatin A, 5 µg/mL aprotinin, and 5 mM DTT) on ice for 30 min. The cells were homogenized and then centrifuged at 1000 g for 10 min at 4°C to remove Nuclei and unbroken cells. The mitochondria fraction was then pelleted by centrifugation at 10,000 g for 20 min at 4°C. The supernatant was centrifuged further at 100,000 g for 60 min at 4°C to get the cytosol fraction.

### Caspase activity assay

The activity of caspases-3 was measured using commercially available caspase colorimetric assay kits (Beyotime, China). Briefly, after the treatment with indicated agents, A459 cells were harvested and washed with PBS by centrifugation at 600 g for 5 min at 4°C. The cell pellets were resuspended in lysis buffer and left on ice for 15 min. The lysates were centrifuged at 160,000 g for 10 min at 4°C and the supernatant was collected for caspase 3 activity assay in the lysis buffer containing Ac-DEVD–pNA according to the kits' instructions. The concentration of pNA was measured at 405 nm with a microtiter plate reader, which used as an indicative of caspase 3 activity.

### Western blot analysis

A549 cells, incubated with the respective conditions, were harvested and lysed. An equal amount of protein was separated by SDS-PAGE (5–15%) and transferred to PVDF membranes. The blotted membranes were blocked with 5% skim milk for 1 h and then incubated with the desired primary antibodies (dilution 1∶1000) overnight at 4°C. Then the immunoreactive bands were visualised by enhanced chemiluminescence using horseradish peroxidase-conjugated IgG secondary antibodies. To quantify equal loading, membrane was reprobed with β-actin antibody.

### Examination of autophagosome by TEM

A549 cells were fixed in 2.5% glutaraldehyde at 4°C overnight, and postfixed with 1% OsO_4_ for 1.5 h. Then cells were stained with 70% ethanol saturated with uranyl acetate, followed by gradient dehydration with ethanol-acetone, and finally embedded in epoxy resin for section. The ultrathin sections were doubly stained by uranyl acetate and lead citrate and analyzed by transmission electron microscopy(TEM)

### RNA interference of Atg7

Atg7 RNA interference was accomplished by transfecting A549 cells with the Atg7-targeted siRNA and the Universal Control siRNA(Invitrogen; 100 pmol/well). Short oligo-RNAs were transfected using Lipofectamine 2000 (Invitrogen) according to the manufacturer's protocol. After 24 h transfection, cells were treated with 5-FU for an additional 48 h. Then cells were collected and cell lysates were subjected to immunoblotting of Atg7,LC3 and PARP. Cells were also processed for cell viability and apoptosis analysis.

### Statistical analysis

The results are expressed as the mean ± SD (n≥3). Statistical analysis between the two groups was calculated using Student's t-test, and multiple groups were performed by SPSS 10.0 software program. *P*<0.05 was considered statistically significant.

## Results

### 5-FU inhibits cell proliferation in A549 cells

**Figure 1:**
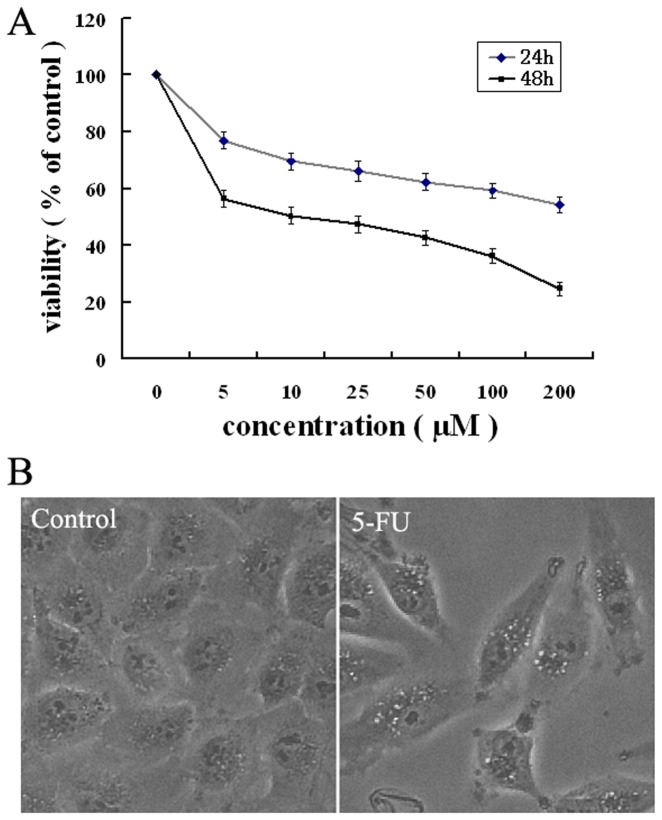
Effect of 5-FU on the viability and morphological changes of A549 cells. (A) Cell viability was determined by MTT assay after treatment with different concentrations of 5-FU for 24 h and 48 h. IC50 was calculated by IC50 software program. (B) Morphological changes was observed after treating cells with 10 µmol/L 5-FU for 48 h by Inverted microscope(Olympus, Japan). All data are representative of at least three independent experiments.

In order to investigate 5-Fluorouracil's potential cell growth inhibition in A549 cells, the effect of 5-FU treatment on cells was examined with a MTT assay. As shown in Fig. 1A,5–FU (0–200 µM) produced a dose- and time- dependent reduction in A549 cell growth. The IC50 for 48 h of 5-FU treatment in A549 cells was 10.32±0.69 µM. Based on the result, 10 µM of 5-FU for 48 h in A549 cells was used for further experiments. In addition, morphological changes also indicated that 5-FU treatment decreased cell density. Interestingly, 5-FU-treated cells did not emerge very obvious apoptotic characters but many vacuoles in the cytoplasm (Fig. 1B). This prompted us to examine whether 5-FU induced autophagy was also included in lung cancer cells.

### 5-FU induces autophagy in A549 cells

**Figure 2:**
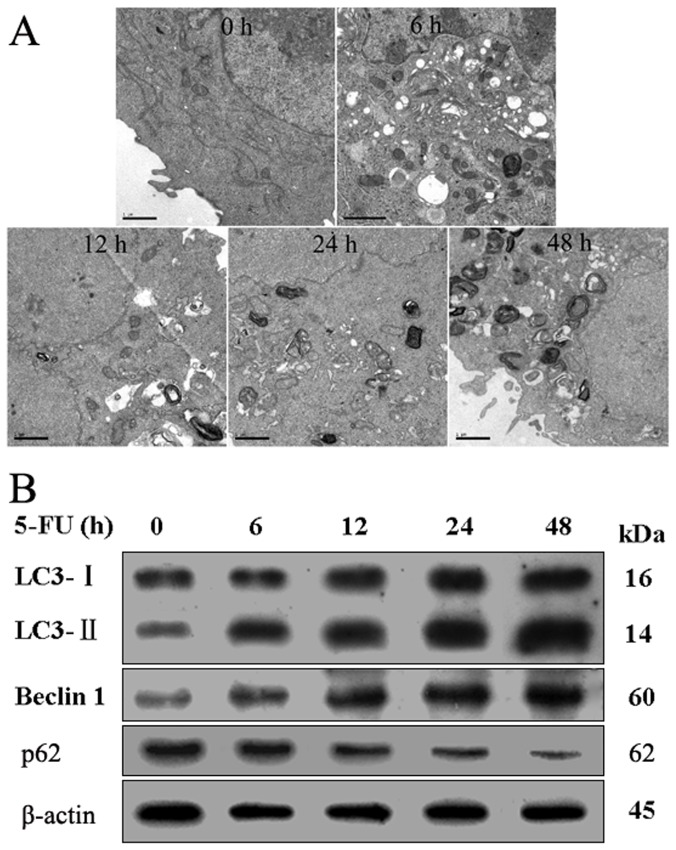
Induction of autophagy in A549 cells by 5-FU treatment. A549 cells were treated with 10 µmol/L 5-FU for different time as indicated, then the autophagosomes and the autophagic levels were detected. (A) The formation of autophagosomes in treated cells were checked by TEM. (B)LC3, Beclin-1 and p62 were examined by western blot. β-actin was a loading control. All data are representative of at least three independent experiments.

Subsequently, transmission electron microscopy was used to check the formation of autophagosomes in 5-FU treated cells. As shown in Figure 2A, treatment with 5-FU for 6–48 h caused the accumulation of autophagic vacuoles, which exhibited autophagosome and/or autolysosomal characteristics, whereas only a few vacuoles were observed in control cells. Autophagy-specific markers LC3, Beclin-1 and p62 were also used to examine the autophagic levels and the autophagic flux in this process by immunoblot analysis. As shown in Fig. 2B, the up-regulation of Beclin-1, the down-regulation of p62 and the conversion of LC3 I/II demonstrated increasing formation of autophagosomes in a time-dependent manner in A549 cells.

**Figure 3:**
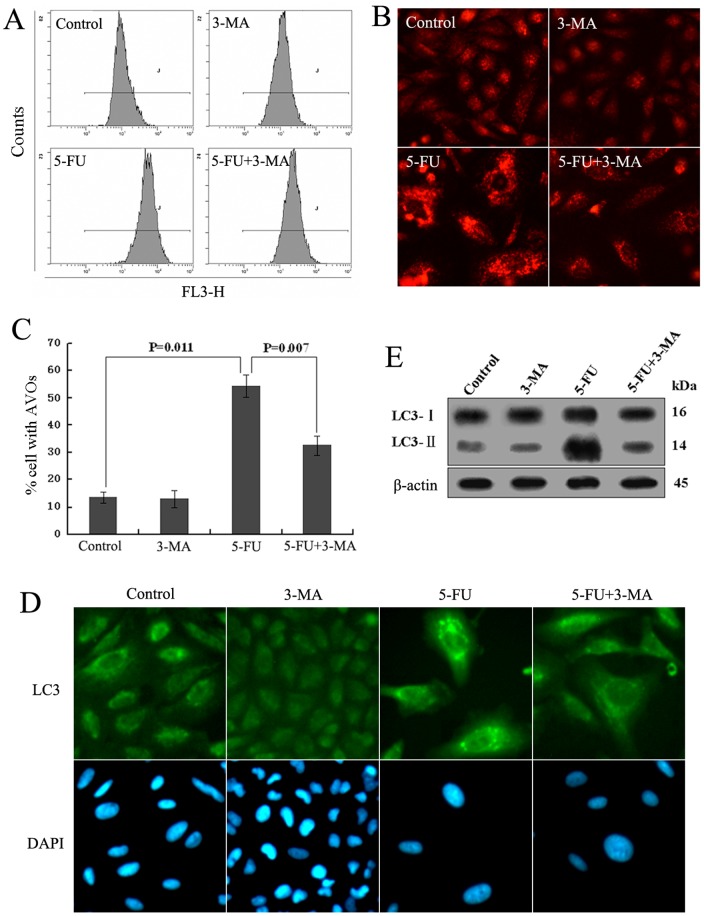
The effect of 3-MA on cell autophagy. Cells were pretreated with 5 mmol/L 3-MA for 1 h before exposure to 10 µmol/L 5-FU for 48 h, then acridine orange was used to stain AVOs, the fluorescence-activated cells were analyzed by flow cytometry (A). After treatment the cells were stained with acridine orange for AVO observation. The cells were visualized under a red filter fluorescence microscope(B). TO quantification of cells developing AVO in A549 cells, the percentage of developed AVOs was calculated based on the results of fluorescence-activated cell sorting assay(C). Fluorescent microscope by immunofluorescence staining for LC3 in the treated-A549 cells(D). Western blot analysis was carried out to detect LC3 protein levels. Blots were re-proved with anti-β-actin as a loading control(E). All data are representative of at least three independent experiments.

Next, In order to further prove the autophagy induced by 5-FU, we introduced 3-MA, which is a popular inhibitor of autophagy [3]. 5 mM 3-MA was added to A549 cells for 1 h prior to 5-FU exposure. Cells were divided into four groups: control (no treatment), 3-MA(treated with 3-MA), 5-FU (treated with 5-FU), and combination (treated with both of 5-FU and 3-MA). Flow cytometry was performed after staining cells with acridine orange for the quantification of AVOs. Results showed that the number of acidic vesicles in 5-FU group increased obviously, which was inhibited by 3-MA, confirming the induction of autophagy (Fig. 3A and C). Similar results were also obtained on fluorescence microscopic examination. As shown in Fig. 3B, A549 cells treated with 5-FU for 48 h displayed a large number of fluorescent vesicles in the cytoplasm, whereas only few of fluorescent vesicles were observed in control group, 3-MA group and combination group. Additionally, LC3 immunofluorescence and immunoblot were also performed. Fluorescence microscopy revealed the punctate distribution of LC3 fluorescence enhanced in 5-FU group (Fig. 3D). Western blot also indicated that the expression of LC3 in combination group partly reverted to the original state, at the same time, reflecting the effective inhibiting effect of 3-MA (Fig. 3E). These results proved that autophagy was induced in 5-FU-treated A549 cells. Therefore, we decided to investigate whether the inhibition of autophagy affects the sensitivity of A549 cell to 5-FU treatment.

### Autophagy inhibition enhances 5-FU-induced apoptotic cell death

**Figure 4:**
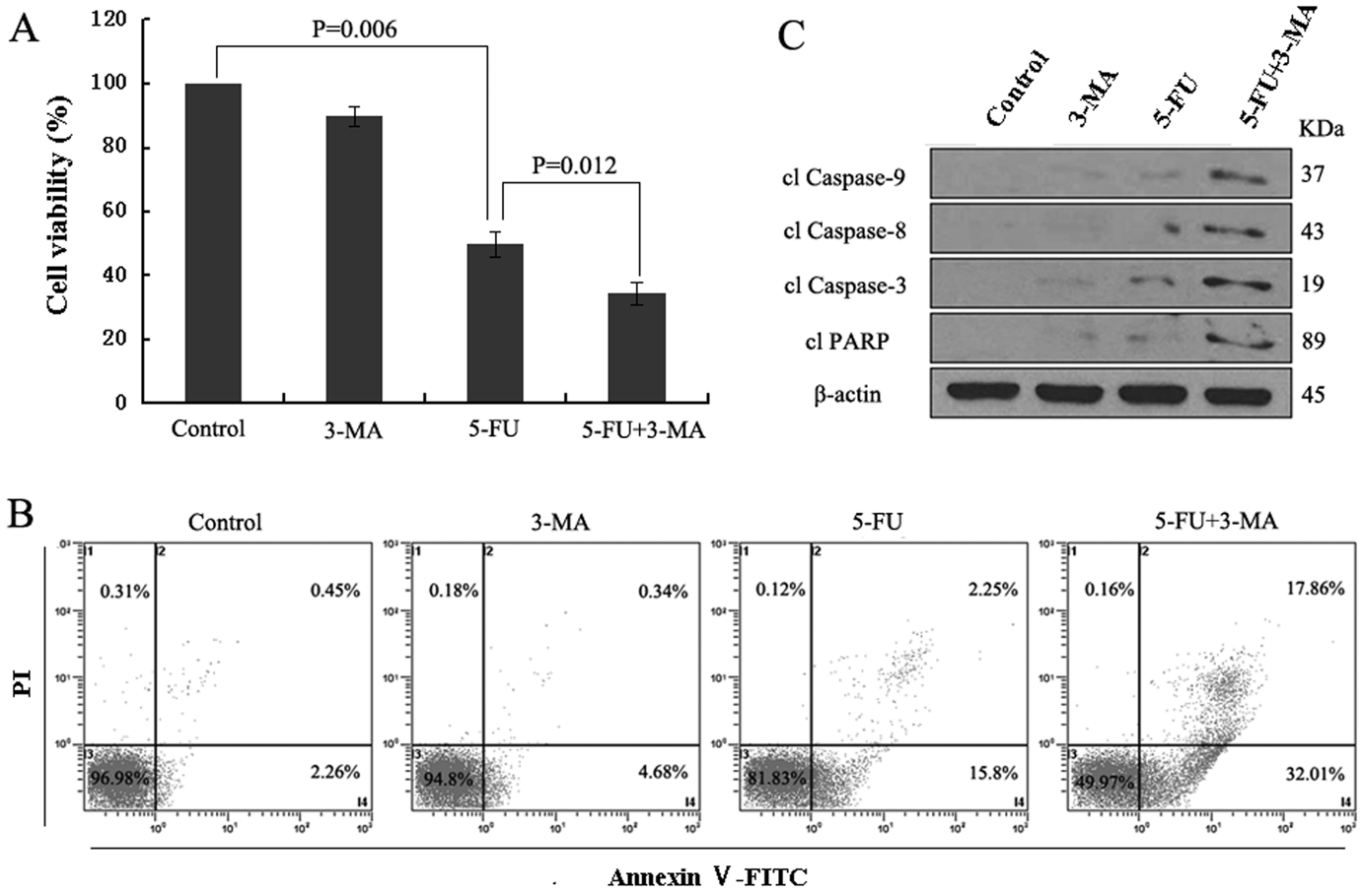
3-MA increases the apoptotic cell death induced by 5-FU. Cells were pretreated with 5 mmol/L 3-MA for 1 h before exposure to 10 µmol/L 5-FU for 48 h. (A) The cell viability was measured with an MTT assay. Data represent means of four independent experiments. (B) Cell death rate was analyzed by the Annexin V assay by flow cytometry as described in the Materials and methods. (C) Cell lysates were prepared and subjected to immunoblotting with antibodies to caspase-9, caspase-8, caspase-3, PARP, and β-actin. All data are representative of at least three independent experiments.

First, to examine whether the inhibition of autophagy sensitizes A549 cells to 5-FU-induced cell death, the effect of treatment was examined with MTT assay (Fig. 4A). In the combination group, the viability of A549 cells decreased faster than in the 5-FU group, the combination group drove 65.75% of the cells to death, about a 39.28% increase over that in the 5-FU group. Although the cell viability in the 3-MA group also decreased, the extent was not significant. These results indicated that 3-MA enhances 5-FU-induced cell death in A549 cells. Next, to validate the observation that inhibition of autophagy affects cell sensitivity to 5-FU, Annexin V–FITC and PI staining was performed. Flow cytometic analysis showed that the number of AV- and AV/PI-positive cells significantly increased in combined treatment group than 5-FU-only treatment group (Fig. 4B). To further confirm this, the executioners, caspase-8, caspase-9, caspase-3 and PARP were then examined. In 5-FU-treated groups, they were all cleaved into their specific active forms, and the activity in the 5-FU-treated cells with inhibited autophagy was significantly higher than in cells treated with 5-FU alone (Fig. 4C).

**Figure 5:**
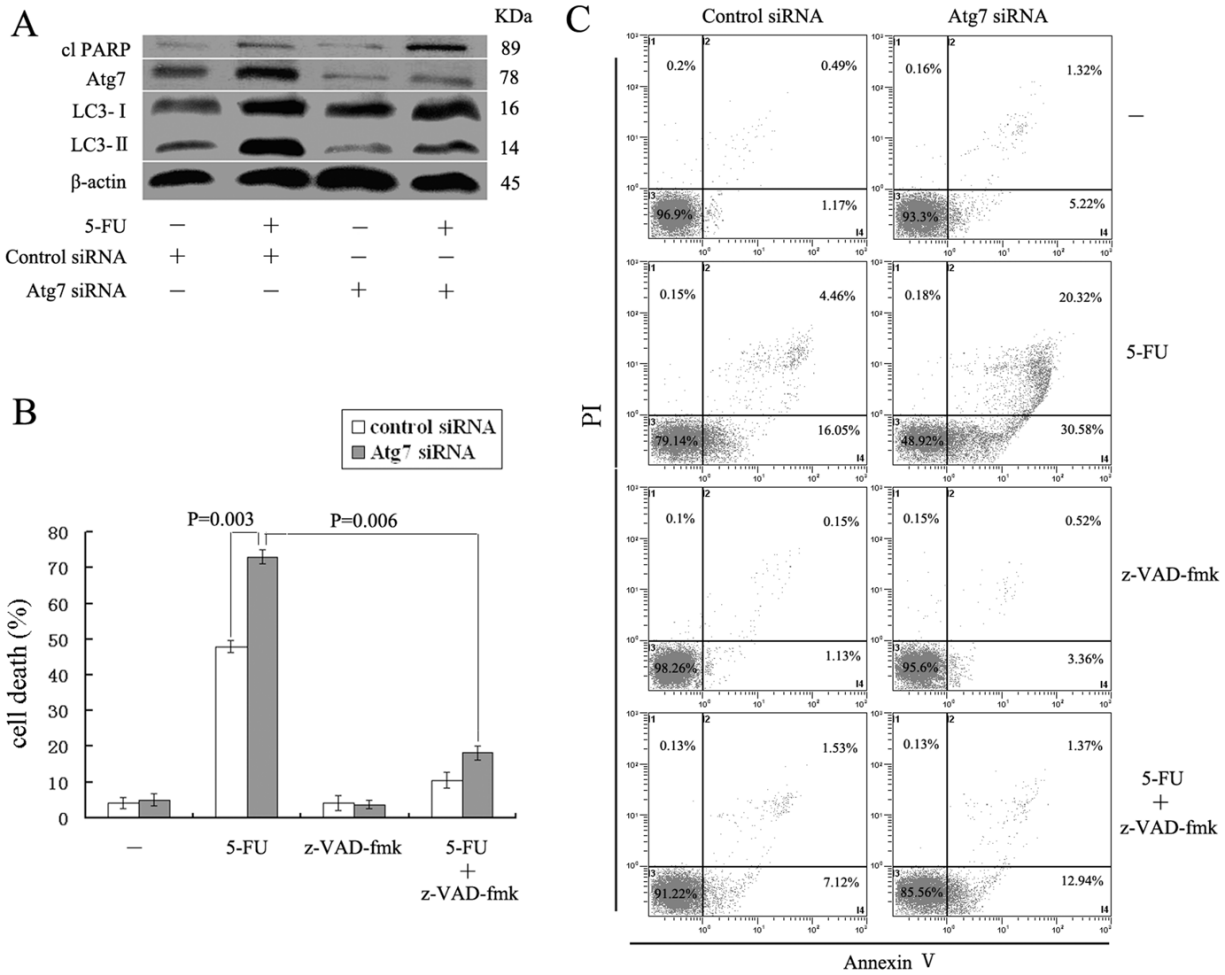
Inhibition of autophagy by Atg7 siRNA increases the apoptotic cell death to 5-FU. Cells were transfected with Atg7-targeted siRNA and the Control siRNA for 24 h before exposure to 10 mmol/L 5-FU for 48 h. (A) Cell lysates were prepared and subjected to immunoblotting with antibodies to LC3, Atg7, PARP, and b-actin. (B) Cell viability was measured using MTT assay. (C) Apoptotic cell death was analyzed by the Annexin V/PI assay. All data are representative of at least three independent experiments.

The role of autophagy in the 5-FU-mediated cytotoxicity was further studied by knocking down the Atg7 expression using siRNA. As shown in Fig. 5, the expression of Atg7 was markedly suppressed in A549 cells transfected with Atg7 siRNA but not those with Control siRNA (Fig. 5A). Accordingly, cells transfected with Atg7 siRNA showed reduced level of LC3-II accumulation and increased level of clPARP after 5-FU treatment (Fig. 5A). In addition, the cytotoxic effect of 5-FU was significantly increased by blocking Atg7 expression and the pancaspase inhibitor z-VAD-fmk rescued the cell death (Fig. 5B). Similarly, the degree of apoptosis induced by 5-FU was also enhanced when knocking down the Atg7 expression and z-VAD-fmk reduced the cell apoptosis significantly (Fig. 5C). As summarized in Fig. 5, knockdown of Atg7 also accelerate the apoptosis induced by 5-FU. These results were consistent with using 3-MA, which demonstrate that autophagy induced by 5-FU plays a protective role of tumor cells against apoptosis, and blockade of autophagy subsequently enhanced the apoptosis through the activation of caspases in A549 cells.

### Changes of mitochondrial membrane potential and release of cytochrome c from mitochondria

**Figure 6:**
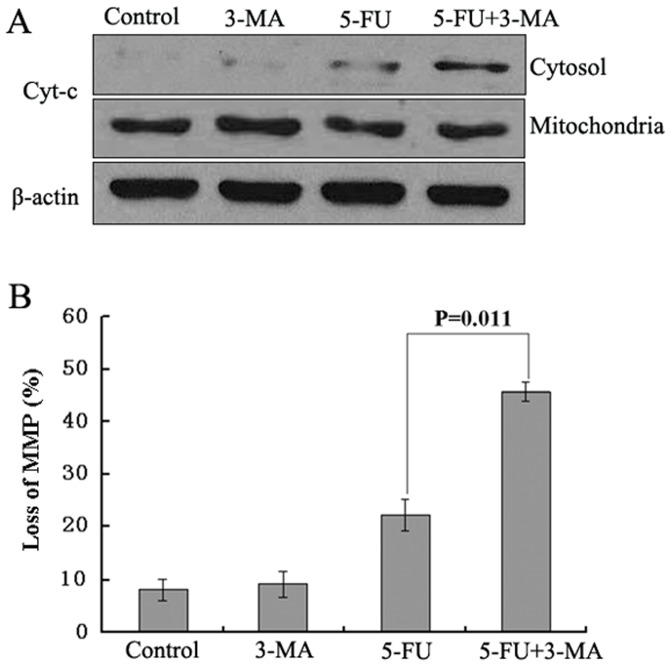
Effect of 3-MA on the cytochrome c release and MMP change by 5-FU treatment. Cells were treated as in Fig. 3. (A)The mitochondria and the cytosol fractions were isolated as described in the Materials and methods, and then were subjected to immunoblotting for the detection of cytochrome c. (B)The MMP change was assessed by JC-1 staining by flow cytometry as described in the Materials and methods. All data are representative of at least three independent experiments.

Previous studies showed that the activation of caspases was driven by cyt-c through intrinsic mitochondrial apoptotic pathway [23], [24]. Therefore, the impact of inhibited autophagy on the 5-FU-mediated release of cyt-c from mitochondria into cytosol was investigated using cell fractionation. The results we obtained revealed that inhibition of autophagy in 5-FU-treated cells led to a drastic increase in the accumulation of cytochrome c in the cytosol (Fig. 6A). The effect on mitochondria was also confirmed by a drop in mitochondrial membrane potential. As shown in Fig. 6B, inhibition of autophagy in 5-FU-treated cells induced an obvious decrease of mitochondrial membrane potential. These data suggest that loss of mitochondrial membrane potential might be required for 5-FU combined 3-MA induced cyt-c release into cytosol, which later triggered the activation and cleavage of caspases and resulted cell apoptosis.

### Inhibition of autophagy stimulates ROS formation, which is required for 5-FU-mediated apoptosis in A549 cells

**Figure 7:**
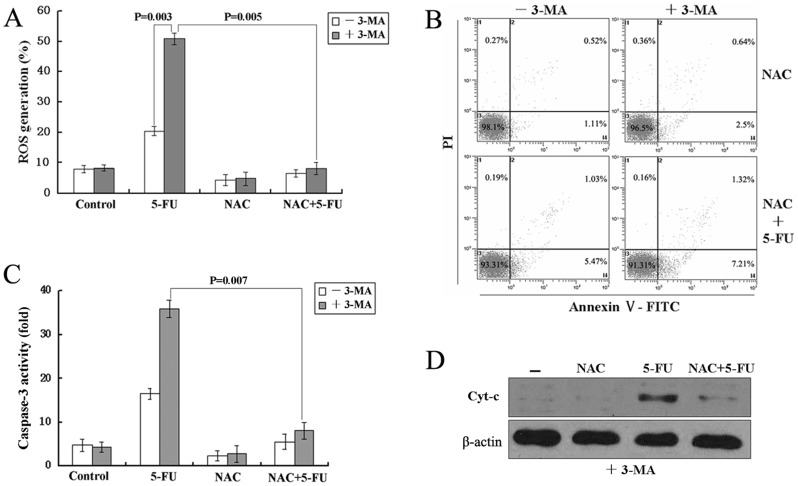
Inhibit autophagy by 3-MA stimulates ROS formation which is required for sensitization of A549 cells to 5-FU-induced apoptosis. Cells were pretreated with 10 mM NAC for 1 h prior to 10 µmol/L 5-FU (48 h) treatment in the presence or absence of 3-MA. (A)The ROS generation was detected using DCFDA by a flow cytometer. Data were processed with the CellQuest software and analyzed by densitometry. (B)Cell death was measured by Annexin V/PI staining. (C)Caspase-3 activity assay was performed as described in the Materials and Methods. (D)The cyt-c level in cytosol fraction was detected by immunoblotting. All data are representative of at least three independent experiments.

Recently, several reports provided evidence for the involvement of reactive oxygen species (ROS) in the induction of autophagy and apoptosis and demonstrated the importance of ROS in the release of cyt-c from mitochondria [25], [26]. Therefore, we decided to analyze whether inhibition of autophagy could stimulate the generation of ROS in 5-FU-treated A549 cells. The treated-cells were stained with chloromethyl-2′,7′-dichlorofluorescein diacetate (CM-H2-DCFDA), a cell permeable fluorescence dye that reacts to a broad spectrum of ROS. As shown in Figure 7A, ROS levels were increased obviously in the cells treated with 5-FU combined with 3-MA compared to 5-FU alone by flow cytometry, and such increase was efficiently attenuated when the cells were pretreated with 10 mM N-acetyl cysteine(NAC) for 1 h. Since the level of ROS was elevated in cells with suppressed autophagy, we further analyzed whether the inhibition of ROS influenced 5-FU-mediated cell death. For this purpose, ROS formation was inhibited by NAC and apoptosis was measured by flow cytometry. As shown in Figure 7B, the sensitization of cells to 5-FU-induced cell apoptosis was completely blocked by preventing ROS formation. Finally, we investigated whether the inhibition of ROS influenced the caspase activity and the release of cyt-c. Indeed, scavenging of ROS inhibited caspase-3 activity, prevented the release of cyt-c from mitochondria, and rescued A549 cells from 5-FU-mediated apoptosis (Fig. 7C and D). These results clearly indicated that the generation of ROS play a major role in regulating cell death in response to 5-FU.

## Discussion

In lung cancer, chemotherapy is widely used to cure and extend postoperative survival in patients. 5-fluorouracil (5-FU) is effectively used as a drug in the treatment of a variety of human cancers, including lung cancer. The major mechanism underlying its antitumor activity has been ascribed to the interference of DNA and RNA synthesis. In the past decades, apoptosis induction by 5-FU has been the major consideration in cancer therapy [27]–[29]. Recently, autophagy has been extensively observed in 5-FU treatment [3], [7], [30]–[32]. In our study, we found that 5-FU could inhibit A549 cell proliferation obviously (Fig. 1A), which included cell autophagy and apoptotic cell death (Fig. 2,3,4 and 5). Our results also showed that both LC3-II and Beclin1, which have been deemed the marker of autophagy [33], was significantly increased after 5-FU treatment in A549 cells accompanied by down-regulation of p62 expression (Fig. 2B). Meanwhile, the increasing formation of autophagosomes and/or autolysosomal with time detected by TEM (Fig. 2A) and the increased formation of characteristic AVOs in cytoplasmic provided another two evidence (Fig. 3A–C). All these results indicated that autophagy was induced by 5-FU.

Autophagy is an intracellular bulk degradation system that is found ubiquitously in eukaryotes, which is responsible for the degradation of most long-lived proteins and some organelles. Cytoplasmic constituents, including organelles, are sequestered into double-membraned autophagosomes and then fuse with lysosomes to form autolysosomes where their contents are degraded. The degraded products are recycled to the cytoplasm for reuse [34]. Autophagy is stimulated in response to external stressors and internal needs to maintain cellular metabolism as well as survival. Nevertheless, autophagy may also result in cell death called “autophagic cell death” (Programmed Cell Death Type II) through excessive self-digestion and degradation of essential cellular constituents under certain circumstances [18], [35]. Recently, a growing body of evidence indicates the role of autophagy in cancer propagation and in response to therapy. However, in cancer chemotherapy, autophagy seems to play a conflicting role, probably promoting or inhibiting apoptosis, in cancer cell survival and death [9]. In order to investigate the contribution of autophagy in the process of 5-FU-induced A549 cell apoptosis, we used autophagy inhibitor 3-MA, a specific inhibitor of the early-stage autophagic process, in this study. As expected, 3-MA inhibited 5-FU-induced autophagy (Fig. 3). Autophagy inhibition enhanced 5-FU-induced cell apoptosis through caspases activation in A549 cells (Fig. 4). To further clarify the role of autophagy in 5-FU-induced A549 cell death, we used Atg7 siRNA to knock down Atg7 and evaluated the role of autophagy more directly. Consistent with the results using 3-MA, transfection of Atg7 siRNA effectively inhibited LC3-II accumulation, increased clPARP production and enhanced the apoptotic cell death induced by 5-FU in A549 cells (Fig. 5). These results suggest that autophagy might play a role as a self-protective mechanism in 5-FU-treated A549 cells and its inhibition may enhance the therapeutic efficacy of 5-FU in the treatment of lung cancer.

However, what is the mechanism of autophagy inhibition sensitizes A549 cell to apoptotic cell death induced by 5-FU? At present, a number of evidence indicates that ROS productions, especially through the mitochondria, are important regulators for autophagy and apoptosis. For instance, the inhibition of autophagy by silencing ATG7 and BECN1 facilitates the stimulation of ROS formation, leading to the sensitization of cells to cisplatin-mediated apoptosis in NSCLC [26]. The inhibition of autophagy with agents such as HCQ, hinders the autophagic protective effect and increases dysfunctional mitochondria and ROS production in prostate cancer cells [36]. In contrast to these studies, suppression of autophagy by silencing ATG7 and ATG8 blocks ROS accumulation and inhibits caspase-independent cell death in macrophages [37]. ROS accumulates after cucurbitacin treatment in Hela cells, and the blocking of this ROS accumulation with two antioxidants NAC and Mito-TEMPO almost completely block the cucurbitacin-induced autophagy and subsequent cell death [38]. In this regard, it is of interest to determine the role of ROS and autophagy in 5-FU-induced A549 cell apoptosis. Our results demonstrated that the inhibition of autophagy led to an increase in ROS formation and the blocking of this ROS accumulation with antioxidant NAC almost completely blocked the 5-FU-induced apoptotic cell death (Fig. 7A and B), suggesting that the accumulation of ROS is an important mechanism in the sensitization of cells to apoptosis under conditions of suppressed autophagy. Furthermore, scavenging of ROS inhibited caspase-3 activity and prevented the release of cyt-c from mitochondria (Fig. 7C and D), which indicated that the apoptotic response to 5-FU treatment in A549 cells with inhibition of autophagy is, to some extent, dependent on the caspase-dependent intrinsic apoptotic pathway. This conclusion was also supported by Fig. 6 and Fig. 5B and C. These results were consistent with an earlier study that autophagy inhibition could enhance the killing efficiency of cisplatin by increasing the formation of ROS that eventually facilitated the permeabilization of the mitochondrial outer membrane, followed by the release of cytochrome c, which subsequently enhanced caspase-dependent apoptosis in NSCLC [26].

In conclusion, our results in A549 cells suggest that 5-FU-induced autophagy might function as a resistance mechanism against apoptotic cell death. Inhibition of autophagy could be a novel strategy for the adjuvant chemotherapy of lung cancer. Furthermore, we demonstrate that the enhancement of apoptosis induced by inhibiting autophagy is regulated by ROS generation, suggesting that regulation of ROS generation and autophagy could provide a powerful strategy to overcome therapy resistance in lung cancer.

## References

[1] Hsin IL, Ou CC, Wu TC, Jan MS, Wu MF, et al (2011) GMI, an immunomodulatory protein from Ganoderma microsporum, induces autophagy in non-small cell lung cancer cells. Autophagy 7 (8) 873–82. 21490426 10.4161/auto.7.8.15698

[2] Liu J, Hu XJ, Jin B, Qu XJ, Hou KZ, et al (2012) β-Elemene induces apoptosis as well as protective autophagy in human non-small-cell lung cancer A549 cells. J Pharm Pharmacol 64: 146–53. 22150682 10.1111/j.2042-7158.2011.01371.x

[3] Li J, Hou N, Faried A, Tsutsumi S, Takeuchi T, et al (2009) Inhibition of autophagy by 3-MA enhances the effect of 5-FU-induced apoptosis in colon cancer cells. Ann Surg Oncol 16: 761–71. 19116755 10.1245/s10434-008-0260-0

[4] Miyake M, Anai S, Fujimoto K, Ohnishi S, Kuwada M, et al (2012) 5-fluorouracil enhances the antitumor effect of sorafenib and sunitinib in a xenograft model of human renal cell carcinoma. Oncol Lett 3: 1195–1202. 22783417 10.3892/ol.2012.662 PMC3392575

[5] Nordman IC, Iyer S, Joshua AM, Clarke SJ (2006) Advance in the adjuvant treatment of colorectal cancer. Anz J Surg 76: 373–80. 16768699 10.1111/j.1445-2197.2006.03726.x

[6] Noro R, Miyanaga A, Minegishi Y, Okano T, Seike M, et al (2010) Histone deacetylase inhibitor enhances sensitivity of non-small-cell lung cancer cells to 5-FU/S-1 via down-regulation of thymidylate synthase expression and uup-regulation of p21^waf1/cip1^ expression. Cancer Sci 101: 1424–30. 20384633 10.1111/j.1349-7006.2010.01559.x PMC11159244

[7] Li J, Hou N, Faried A, Tsutsumi S, Kuwano H (2010) Inhibition of autophagy augments 5-fluorouracil chemotherapy in human colon cancer in vitro and in vivo model. Eur J Cancer 46: 1900–9. 20231086 10.1016/j.ejca.2010.02.021

[8] Yi H, Cho HJ, Cho SM, Jo K, Park JA, et al (2012) Effect of 5-FU and MTX on the Expression of Drug-resistance Related Cancer Stem Cell Markers in Non-small Cell Lung Cancer Cells. Korean J Physiol Pharmacol 16: 11–6. 22416214 10.4196/kjpp.2012.16.1.11 PMC3298820

[9] Xi G, Hu X, Wu B, Jiang H, Young CY, Pang Y, Yuan H (2011) Autophagy inhibition promotes paclitaxel-induced apoptosis in cancer cells. Cancer Lett 307: 141–8. 21511395 10.1016/j.canlet.2011.03.026

[10] Liu D, Yang Y, Liu Q, Wang J (2011) Inhibition of autophagy by 3-MA potentiates cisplatin-induced apoptosis in esophageal squamous cell carcinoma cells. Med Oncol 28: 105–11. 20041317 10.1007/s12032-009-9397-3

[11] Sharma N, Thomas S, Golden EB, Hofman FM, Chen TC, et al (2012) Inhibition of autophagy and induction of breast cancer cell death by mefloquine, an antimalarial agent. Cancer Lett 326: 143–54. 22863539 10.1016/j.canlet.2012.07.029

[12] Cui Q, Tashiro S, Onodera S, Minami M, Ikejima T (2007) Autophagy preceded apoptosis in oridonin-treated human breast cancer MCF-7 cells. Biol Pharm Bull. 30: 859–64. 10.1248/bpb.30.859 17473426

[13] Li X, Wu WK, Sun B, Cui M, Liu S, et al (2011) Dihydroptychantol A, a macrocyclic bisbibenzyl derivative, induces autophagy and following apoptosis associated with p53 pathway in human osteosarcoma U2OS cells. Toxicol Appl Pharmacol 251: 146–54. 21185854 10.1016/j.taap.2010.12.007

[14] Maiuri MC, Zalckvar E, Kimchi A, Kroemer G (2007) Self-eating and self-killing: crosstalk between autophagy and apoptosis. Nat Rev Mol Cell Biol 8: 741–752. 17717517 10.1038/nrm2239

[15] Jiang H, White EJ, Conrad C, Gomez-Manzano C, Fueyo J (2009) Autophagy pathways in glioblastoma. Methods Enzymol 453: 273–86. 19216911 10.1016/S0076-6879(08)04013-5

[16] Ling YH, Aracil M, Zou Y, Yuan Z, Lu B, et al (2011) PM02734 (elisidepsin) induces caspase-independent cell death associated with features of autophagy, inhibition of the Akt/mTOR signaling pathway, and activation of death-associated protein kinase. Clin Cancer Res 17: 5353–66. 21690574 10.1158/1078-0432.CCR-10-1948

[17] D'Autreaux B, Toledano MB (2007) ROS as signaling molecules:mechanisms that generate specificity in ROS homeostasis. Nat Rev Mol Cell Biol 8: 813–24. 17848967 10.1038/nrm2256

[18] Dewaele M, Maes H, Agostinis P (2010) ROS-mediated mechanisms of autophagy stimulation and their relevance in cancer therapy. Autophagy 6: 838–54. 20505317 10.4161/auto.6.7.12113

[19] Nicolau-Galmés F, Asumendi A, Alonso-Tejerina E, Pérez-Yarza G, Jangi SM, et al (2011) Terfenadine induces apoptosis and autophagy in melanoma cells through ROS-dependent and -independent mechanisms. Apoptosis 16: 1253–67. 21861192 10.1007/s10495-011-0640-y PMC3204001

[20] Bellot GL, Liu D, Pervaiz S (2012) ROS, autophagy, mitochondria and cancer: Ras, the hidden master? Mitochondrion [Epub ahead of print]. 10.1016/j.mito.2012.06.007 22750269

[21] Yoon S, Woo SU, Kang JH, Kim K, Kwon MH, et al (2010) STAT3 transcriptional factor activated by reactive oxygen species induces IL6 in starvation-induced autophagy of cancer cells. Autophagy 6: 1125–38. 20930550 10.4161/auto.6.8.13547

[22] Pan XH, Liu X, Zhao BX, Xie YS, Shin DS, et al (2008) 5-Alkyl-2-ferrocenyl-6,7-dihydropyrazolo[1,5-a]pyrazin-4(5H)-one derivatives inhibit growth of lung cancer A549 cell by inducing apoptosis. Bioorg Med Chem 16: 9093–100. 18829332 10.1016/j.bmc.2008.09.046

[23] Lin HI, Lee YJ, Chen BF, Tsai MC, Lu JL, et al (2005) Involvement of Bcl-2 family, cytochrome c and caspase 3 in induction of apoptosis by beauvericin in human non-small cell lung cancer cells. Cancer Lett 230: 248–59. 16297711 10.1016/j.canlet.2004.12.044

[24] Gallego MA, Joseph B, Hemström TH, Tamiji S, Mortier L, et al (2004) Apoptosis-inducing factor determines the chemoresistance of non-small-cell lung carcinomas. Oncogene 23: 6282–91. 15286713 10.1038/sj.onc.1207835

[25] Macchioni L, Davidescu M, Sciaccaluga M, Marchetti C, Migliorati G, et al (2011) Mitochondrial dysfunction and effect of antiglycolytic bromopyruvic acid in GL15 glioblastoma cells. J Bioenerg Biomembr 43: 507–18. 21833601 10.1007/s10863-011-9375-2

[26] Kaminskyy V, Piskunova T, Zborovskaya I, Tchevkina E, Zhivotovsky B (2012) Suppression of basal autophagy reduces lung cancer cell proliferation and enhances caspase-dependent and -independent apoptosis by stimulating ROS formation. Autophagy 1: 8 (7). 10.4161/auto.20123 PMC3429541 22562073

[27] Chen CY, Jia JH, Zhang MX, Zhou YB, Zhang RM, et al (2006) Comparative proteomics of apoptosis initiation induced by 5-fluorouracil in human gastric cancer. Chin J Physiol 49: 31–8. 16900703

[28] Qin L, Zhang X, Zhang L, Feng Y, Weng GX, et al (2008) Downregulation of BMI-1 enhances 5-fluorouracil-induced apoptosis in nasopharyngeal carcinoma cells. Biochem Biophys Res Commun 371: 531–5. 18452707 10.1016/j.bbrc.2008.04.117

[29] Wang CZ, Li XL, Sun S, Xie JT, Aung HH, et al (2009) Methylnaltrexone, a peripherally acting opioid receptor antagonist, enhances tumoricidal effects of 5-Fu on human carcinoma cells. Anticancer Res 29: 2927–32. 19661297

[30] Xiong HY, Guo XL, Bu XX, Zhang SS, Ma NN, et al (2010) Autophagic cell death induced by 5-FU in Bax or PUMA deficient human colon cancer cell. Cancer Lett 288: 68–74. 19660860 10.1016/j.canlet.2009.06.039

[31] Sasaki K, Tsuno NH, Sunami E, Tsurita G, Kawai K, et al (2010) Chloroquine potentiates the anti-cancer effect of 5-fluorouracil on colon cancer cells. BMC Cancer 10: 370. 20630104 10.1186/1471-2407-10-370 PMC2914703

[32] Nyhan MJ, O'Donovan TR, Elzinga B, Crowley LC, O'Sullivan GC, et al (2012) The BH3 mimetic HA14-1 enhances 5-fluorouracil-induced autophagy and type II cell death in oesophageal cancer cells. Br J Cancer 106: 711–8. 22240779 10.1038/bjc.2011.604 PMC3322956

[33] Klionsky DJ, Abdalla FC, Abeliovich H, Abraham RT, Acevedo-Arozena A, et al (2008) Guidelines for the use and interpretation of assays for monitoring autophagy in higher eukaryotes. Autophagy 4: 151–75. 18188003 10.4161/auto.5338 PMC2654259

[34] Mizushima N (2004) Methods for monitoring autophagy. Int J Biochem Cell Biol 36: 2491–502. 15325587 10.1016/j.biocel.2004.02.005

[35] Galluzzi L, Vicencio JM, Kepp O, Tasdemir E, Maiuri MC, et al (2008) To die or not to die: That is the autophagic question. Curr Mol 8: 78–91. 10.2174/156652408783769616 18336289

[36] Saleem A, Dvorzhinski D, Santanam U, Mathew R, Bray K, et al (2012) Effect of dual inhibition of apoptosis and autophagy in prostate cancer. Prostate 72: 1374–81. 22241682 10.1002/pros.22487 PMC3840901

[37] Xu Y, Kim SO, Li Y, Han J (2006) Autophagy contributes to caspase-independent macrophage cell death. J Biol Chem 281: 19179–87. 16702227 10.1074/jbc.M513377200

[38] Zhang T, Li Y, Park KA, Byun HS, Won M, et al (2012) Cucurbitacin induces autophagy through mitochondrial ROS production which counteracts to limit caspase-dependent apoptosis. Autophagy 8: 559–76. 22441021 10.4161/auto.18867

